# Outcomes in patients with aortic stenosis and severely reduced ejection fraction following surgical aortic valve replacement and transcatheter aortic valve replacement

**DOI:** 10.1186/s13019-024-02724-9

**Published:** 2024-04-20

**Authors:** Eric R. Bain, Bistees George, Syed H. Jafri, Roopa A. Rao, Anjan K. Sinha, Maya E. Guglin

**Affiliations:** 1 Department of Internal Medicine, Indiana University School of Medicine, 635 Barnhill Drive Van Nuys Medical Science Building 116, Indianapolis, IN 46202 USA; 2 Indiana University School of Medicine, Krannert Institute of Cardiology, Indianapolis, USA

**Keywords:** Aortic valve, Stenosis, Valve replacement, Ejection fraction, Mortality

## Abstract

**Background:**

Patients with severe aortic stenosis (AS) and left ventricular (LV) dysfunction demonstrate improvement in left ventricular injection fraction (LVEF) after aortic valve replacement (AVR). The timing and magnitude of recovery in patients with very low LVEF (≤ 25%) in surgical or transcatheter AVR is not well studied.

**Objective:**

Determine clinical outcomes following transcatheter aortic valve replacement (TAVR) and surgical aortic valve repair (SAVR) in the subset of patients with severely reduced EF ≤ 25%.

**Methods:**

Single-center, retrospective study with primary endpoint of LVEF 1-week following either procedure. Secondary outcomes included 30-day mortality and delayed postprocedural LVEF. T-test was used to compare variables and linear regression was used to adjust differences among baseline variables.

**Results:**

83 patients were enrolled (TAVR = 56 and SAVR = 27). TAVR patients were older at the time of procedure (TAVR 77.29 ± 8.69 vs. SAVR 65.41 ± 10.05, *p* < 0.001). One week post procedure, all patients had improved LVEF after both procedures (*p* < 0.001). There was no significant difference in LVEF between either group (TAVR 33.5 ± 11.77 vs. SAVR 35.3 ± 13.57, *p* = 0.60). Average LVEF continued to rise and increased by 101% at final follow-up (41.26 ± 13.70). 30-day mortality rates in SAVR and TAVR were similar (7.4% vs. 7.1%, *p* = 0.91).

**Conclusion:**

Patients with severe AS and LVEF ≤ 25% have a significant recovery in post-procedural EF following AVR regardless of method. LVEF doubled at two years post-procedure. There was no significant difference in 30-day mortality or mean EF recovery between TAVR and SAVR.

**Trial registration:**

Indiana University institutional review board granted approval for above study numbered 15,322.

## Background

Aortic stenosis (AS) is currently the second most common valvular disease in the United States and its prevalence is expected to grow [1]. In patients with severe AS there is a significant increase in 5-year mortality without intervention, and it is a class I recommendation for aortic valve replacement (AVR) [2, 3]. Surgical aortic valve replacement (SAVR) has previously been the gold standard; however, transcatheter aortic valve replacement (TAVR) has grown to be a more favorable, less invasive alternative for the spectrum of high surgical risk candidates [4–7].

Impaired left ventricular (LV) function is associated with worse outcomes in patients with severe AS, and treatment with AVR has been shown to have survival benefit [8–11]. This subset of patients has increased mortality following SAVR, and there is conflicting data on risk associated with TAVR [8, 9, 12–15]. Recent studies have shown that patients with AS and mild to moderately reduced left ventricular ejection fraction (LVEF) (35–50%) have improved LVEF and similarly reduced mortality after SAVR or TAVR. It remains unclear in the subset of patients with severely reduced EF if there is a superior treatment choice [4].

This study seeks to delineate the natural history of patients with severely reduced EF (≤ 25%) and severe AS following TAVR versus SAVR.

## Methods

A retrospective review was performed of all patients who underwent TAVR or SAVR at a single institution between January 2012 to December 2020. Echocardiogram data were reviewed and only patients with both severe AS and severely reduced EF (≤ 25%) were included. Severe AS was defined by a mean transvalvular gradient > 40 mmHg, peak jet velocity > 4.0 m/s, or valve area < 1${\text{c}\text{m}}^{2},$ assessed by echocardiography with the patient at rest. Patient baseline data were collected prior to the procedure. Post-procedure follow-up data was reviewed at the following intervals: 1 week, 6 months, 6–12 months, 12–18 months, 18–24 months, and > 24 months.

The study was granted exempt by the Indiana University institutional review board, and written consent was waived.

### Endpoints

The primary endpoint was LVEF one week post procedure. Secondary outcomes included 30-day mortality, delayed post procedure LVEF (6-month, 12-month, 24-month), and postprocedural changes in echocardiographic variables (aortic valve area, left ventricular end-diastolic diameter, left ventricular end-systolic diameter, aortic valve peak velocity, and aortic valve mean gradient). These variables were tested hierarchically for noninferiority or superiority. Groups were compared based on the type of procedure (TAVR vs. SAVR) at the time intervals listed above.

### Procedures

Pre-procedure doppler echocardiographic data was collected for each subject from TTE or TEE reports including LV function, LV dimensions, and valve function.

For patients who had multiple pre-procedure echocardiographic studies, the study obtained closest to the TAVR/SAVR was utilized. If the most recent echocardiogram lacked specific variables, these values were supplemented from the next most recent echocardiogram.

### Statistics

SPSS was used for statistical analysis. *P* values < 0.05 were considered statistically significant. Continuous variables were reported as mean ± standard deviation (SD) and were compared using students T-test as all data was parametric. Categorical variables were described as frequency and percentages and were compared with Fisher exact test.

Time to event end points were presented as Kaplan- Meier estimates and were compared using log-rank test using Stata v16.1. Linear regression was used to adjust differences among baseline variables. Multivariate logistic regression model was used to compare 30-day mortality outcomes. The model adjusted for clinically relevant variables including age, aortic valve method, and smoking history.

## Results

Eighty-three patients were identified that met study criteria. 56 (67.4%) underwent TAVR 27 (32.5%) underwent SAVR. Demographic and baseline characteristics including pre-procedure echocardiogram parameters are shown in Tables 1 and 2, respectively. The mean (SD) age was 73 (10.7) years and 56 (67%) were male. The average pre-procedure LVEF was 20.5% (4.9). A total of 40 patients (48.19%) had an LVEF less than or equal to 20.

**Table 1:** Patient demographics and characteristics

Variable	All Patients *N* = 83 (%)	TAVR *N* = 56 (67%)	SAVR *N* = 27 (32%)		*P*- Value
Age, mean (SD)	73.4 (10.7)	77.29 (8.69)	65.41 (10.05)		< 0.001
Gender, n (%)					0.79
Male	60 (72.3%)	41 (73.2%)	19 (70.4%)		
Female	23 (27.7%)	15 (26.8%)	8 (29.6%)		
Race**, n (%)					0.99
White	79 (96.3%)	53 (96.4%)	26 (96.3%)		
Black	3 (3.7%)	2 (3.6%)	1 (3.7%)		
Weight, mean (SD)	84.06 (23.4)	81.53 (22.56)	89.29 (24.7)		0.16
BMI, mean (SD)	28.3 (7.1)	27.35 (6.84)	30.19 (7.52)		0.09
Medical History, n (%)					
Smoking	17 (20.5%)	10 (18%)	7 (25.9%)		0.00
PVD	25 (30.1%)	21 (37.5%)	4 (14.8%)		0.04
MI	24 (28.9%)	21 (37.5%)	3 (11.1%)		0.21
HTN	65 (78.3%)	43 (76.8%)	22 (81.5%)		0.63
DM	43 (51.8%)	27 (48.2%)	16 (59.3%)		0.59
Atrial fibrillation/flutter	25 (30.1%)	18 (32.1%)	7 (25.9%)		0.57
Paroxysmal	13 (15.7%)	7 (29.2%)	6 (22.2%)		
Persistent	8 (9.6%)	7 (29.2%)	1 (3.7%)		
Not listed	4 (4.8%)	4 (16.7%)	0 (0%)		
NYHA HF Class, n(%)	79	55	24		0.35
I	2 (2.4%)	1 (1.8%)	1 (3.7%)		0.55
II	7 (8.4%)	3 (5.4%)	4 (14.8%)		0.11
III	35 (42.2%)	23 (41.1%)	12 (44.4%)		0.51
IV	35 (42.2%)	28 (50.0%)	7 (25.9%)		0.08
Procedure History, n(%)					
Percutaneous Coronary Intervention	27 (32.5%)	25 (44.6%)	2 (7.4%)		0.001
CABG	21 (25.3%)	19 (33.9%)	2 (7.4%)		0.009
PPM	7 (8.4%)	7 (12.5%)	0 (0%)		0.056
ICD	17 (20.5%)	15 (26.8%)	2 (7.4%)		0.048
Aortic valve Procedure	16 (19.3%)	13 (23.2%)	3 (11.1%)		0.195
Aortic valve replacement	3 (3.6%)	3 (5.4%)	0 (0%)		0.390
Aortic valve balloon angioplasty	13 (15.7%)	10 (17.9%)	3 (11.1%)		0.390
Non-Aortic valve procedure	3 (3.6%)	2 (3.6%)	1 (3.7%)		0.976

*Categorical variables compared using Fischers Exact Test

Continuous variables compared using Students T-Test

** 1 patient excluded due to reporting other

^ SD-Standard Deviation, PVD-Peripheral Vascular Disease, MI-Mycoardial infarction, HTN-Hypertension, DM-Diabetes Mellitus, NYHA FS – New York Heart Association Functional Score, CABG- Coronary Artery Bypass Graft Surgery, PPM-Permanent Pacemaker, ICD-Implantable Cardioverter Defibrillator

**Table 2:** Echo parameters pre and post procedure

Variable		All Patients (*n* = 83)		SAVR (*n* = 27)		TAVR (*n* = 56)			*P*-Value				
PRE PROCEDURE ECHO DATA (*n* = 83)													
LVEF, mean (SD)		20.48 (4.29)		20.67 (3.73)		20.39 (4.58)			0.79				
AV Peak Velocity (m/s), mean (SD)		3.8722 (3.4)		3.60 (0.669)		4.00 (4.15)			0.62				
AV Area, mean (SD)		0.66 (0.19)		0.6944 (0.22365)		0.648 (0.168)			0.3				
AV Mean Gradient (mmHg), mean (SD)		28.32 (11.69)		30.736 (12.47)		27.241 (11.26)			0.22				
AV Peak Gradient, mean (SD)		49.09 (20.22)		48.76 (20.88)		49.25 (20.11)			0.92				
LV Septal Thickness, mean (SD)		1.19 (0.267)		1.275 (0.255)		1.15 (0.265)			0.059				
Posterior wall thickness, mean (SD)		1.15 (0.217)		1.23 (0.205)		1.11 (0.215)			0.03				
1 WEEK POST PROCEDURE ECHO DATA (*n* = 64)													
LVEF, mean (SD)		33.5 (11.77)		35.3 (13.57)		33.17 (11.52)			0.60				
AV Peak Velocity (m/s), mean (SD)		1.98 (0.534)		2.5 (0.60)		1.9 (0.48)			0.002				
AV Area, mean (SD)		1.81 (0.550)		*		1.81 (0.55)			N/A				
AV Mean Gradient (mmHg), mean (SD)		8.09 (4.814)		12.24 (6.5)		7.54 (4.33)			0.01				
LV Septal Thickness, mean (SD)		1.18 (0.234)		1.23 (0.17)		1.17 (0.24)			0.48				
Posterior wall thickness, mean (SD)		1.22 (0.230)		1.28 (0.26)		1.27 (0.23)			0.36				
6 MONTH POST PROCEDURE ECHO DATA (*n* = 45)													
LVEF, mean (SD)		38.87 (14.26)		38.92 (9.69)		36.03 (15.80)			0.54				
AV Peak Velocity (m/s), mean (SD)		2.07 (0.535)		2.44 (0.482)		1.936 (0.4936)			0.01				
AV Area, mean (SD)		1.71 (0.651)		1.22 (0.380)		1.82 (0.654)			0.09				
AV Mean Gradient (mmHg), mean (SD)		8.94 (4.75)		11.52 (4.61)		7.80 (4.43)			0.03				
LV Septal Thickness, mean (SD)		1.18 (0.225)		1.27 (0.215)		1.15 (0.223)			0.09				
Posterior wall thickness, mean (SD)		1.16 (0.24)		1.27 (0.222)		1.121 (0.239)			0.055				
12 MONTH POST PROCEDURE ECHO DATA (*n* = 21)													
LVEF, mean (SD)		36.9 (12.22)		33.20 (6.30)		38.06 (13.5)			0.45				
AV Peak Velocity (m/s), mean (SD)		2.37 (1.16)		2.16 (0.632)		2.46 (1.33)			0.64				
AV Area, mean (SD)		1.65 (0.78)		1.35 (0.353)		1.72 (0.858)			0.58				
AV Mean Gradient (mmHg), mean (SD)		9.89 (6.12)		11.88 (9.42)		9.16 (4.87)			0.47				
LV Septal Thickness, mean (SD)		1.23 (0.24)		1.24 (0.114)		1.23 (0.274)			0.92				
Posterior wall thickness, mean (SD)		1.17 (0.19)		1.22 (0.837)		1.16 (0.218)			0.54				
18 MONTH POST PROCEDURE ECHO DATA (*n* = 14)													
LVEF, mean (SD)		39.57 (16.86)		51.8 (13.27)		32.78 (15.11)			0.04				
AV Peak Velocity (m/s), mean (SD)		1.50 (0.43)		1.32 (0.48)		1.60 (0.42)			0.43				
AV Mean Gradient (mmHg), mean (SD)		2.00 (0.67)		5.08 (2.78)		8.16 (5.2)			0.33				
LV Septal Thickness, mean (SD)		1.27 (0.22)		1.23 (0.27)		1.29 (0.38)			0.78				
Posterior wall thickness, mean (SD)		1.22 (0.25)		1.30 (0.33)		1.17 (0.20)			0.39				
24 MONTH POST PROCEDURE ECHO DATA (*n* = 14)													
LVEF, mean (SD)		35.07 (16.04)		36.1 (14.5)		32.5 (21.8)			0.72				
AV Peak Velocity (m/s), mean (SD)		2.51 (0.57)		2.48 (0.65)		2.59 (0.43)			0.77				
AV Area, mean (SD)		1.20 (0.43)		1.27 (0.54)		1.10 (0.212)			0.58				
AV Mean Gradient (mmHg), mean (SD)		14.13 (7.07)		13.4 (6.82)		15.58 (8.41)			0.64				
LV Septal Thickness, mean (SD)		1.27 (0.32)		1.36 (0.316)		1.035 (0.211)			0.09				
Posterior wall thickness, mean (SD)		1.26 (0.26)		1.33 (0.27)		1.11 (0.155)			0.16				
24 + MONTH POST PROCEDURE ECHO DATA (*n* = 23)													
LVEF, mean (SD)		41.26 (13.70)		39.75 (11.8)		42.91 (15.9)			0.59				
AV Peak Velocity (m/s), mean (SD)		2.28 (0.46)		2.41 (0.52)		2.12 (0.35)			0.17				
AV Mean Gradient (mmHg), mean (SD)		11.44 (5.17)		13.23 (6.06)		9.66 (3.50)			0.16				
LV Septal Thickness, mean (SD)		1.23 (0.25)		1.29 (0.22)		1.15 (0.27)			0.19				
Posterior wall thickness, mean (SD)		1.19 (0.22)		1.12 (0.19)		1.17 (0.27)			0.66				

*Categorical variables compared using Fischers Exact Test

Continuous variables compared using Students T-Test

^ SD-Standard Deviation, LVEF-Left Ventricular Ejection Fraction, AV-Aortic Valve

TAVR patients were significantly older 77 vs. 65 at time of AVR (*p* < 0.001) with higher rates of peripheral vascular disease (38% vs. 15%, *p* = 0.035). TAVR patients also had higher rates of prior procedures including percutaneous coronary intervention (45% vs. 8%, *p* < 0.001), coronary artery bypass graft surgery (34% vs. 7%, *p* < 0.009) and implantable cardioverter-defibrillator placement (27% vs. 7%, *p* = 0.048). On preprocedural echocardiogram SAVR patients had a greater posterior wall thickness (1.23 vs. 1.11 *p* = 0.03) and increased septal wall thickness which neared significance (1.27 vs. 1.15, *p* = 0.06). Other preoperative Echo characteristics were similar between groups.

**Fig. 1:**
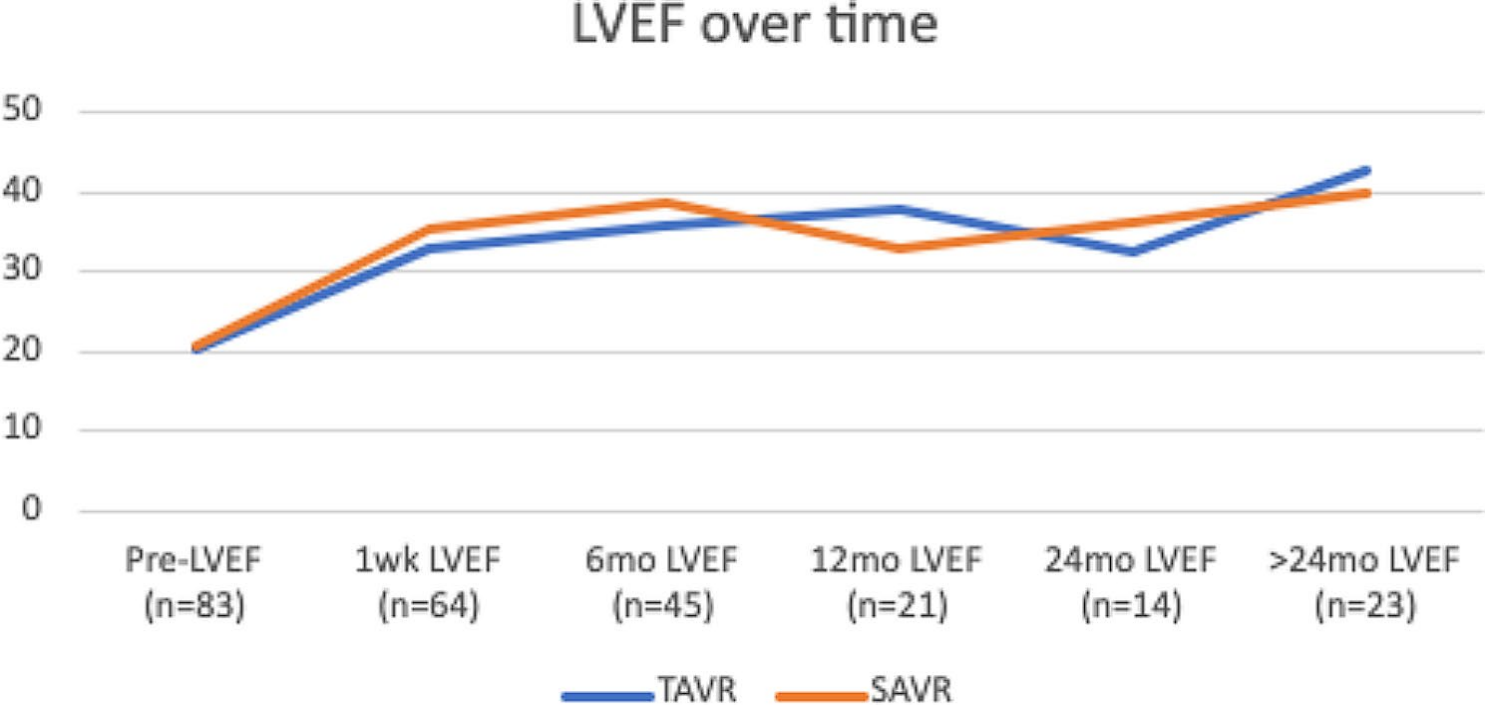
Comparison of pre-procedural EF (TAVR and SAVR combined) vs post-procedural EF at 1 week, 6 months, 12 months, 18 months, 24 months, and > 2 years post procedure

Post procedure echocardiogram parameters (Table 2) were similar between groups. LVEF improved significantly after both procedures at all time points as shown in Table 3. The average LVEF at 1 week post procedure increased by 60% for an average LVEF of 33.5. LVEF continued to increase up to last follow-up (> 24 months) at which time the average LVEF was 41.26, a 101% average increase (Table 4). There was no significant difference between the change in LVEF between TAVR and SAVR patients (Fig. 1).

**Table 3:** Comparison of pre-procedural EF (TAVR and SAVR combined) vs. post-procedural EF at 1 week, 6 months, 12 months, 18 months, 24 months, and > 2 years post procedure

Left Ventricular Ejection Fraction	Total Patients (*N* = 83)	Mean (SD)	95% CI	*P*-Value
Pre Procedure	83 (100%)	20.48 (4.298)	(19.54–21.42)	*p* < 0.001
1 week Post	64 (77%)	33.50 (11.77)	(30.56–36.44)	
6 months Post	45 (54%)	36.87 (14.26)	(32.58–41.15)	
12 months Post	21 (25.3%)	36.90 (12.218)	(31.34–42.47)	
18 months Post	14 (16.9%)	39.57 (16.856)	(29.84–49.30)	
24 months Post	14 (16.9%)	35.07 (16.036)	(25.81–44.33)	
> 2years Post	23 (27.7%)	41.26 (13.695)	(35.34–47.18)	

**Table 4:** Change in LVEF post-procedure

Change in LVEF (time from procedure)	All Patients	TAVR (%)	SAVR (%)	*P*-Value
1 week	13.02 (64%)	12.2 (59.8%)	13.6 (65.8%)	0.758
6 months	18.39 (90%)	16 (78.5%)	17.9 (86.6%)	0.966
12 months	16.42 (80%)	15.9 (78.0%)	16.2 (78.4%)	0.966
24 months	14.59 (71%)	12.3 (60.3%)	16.4 (79.3%)	0.7
> 24 months	20.78 (101%)	22.1 (108.4%)	19.2 (92.9%)	0.662

**Fig. 2:**
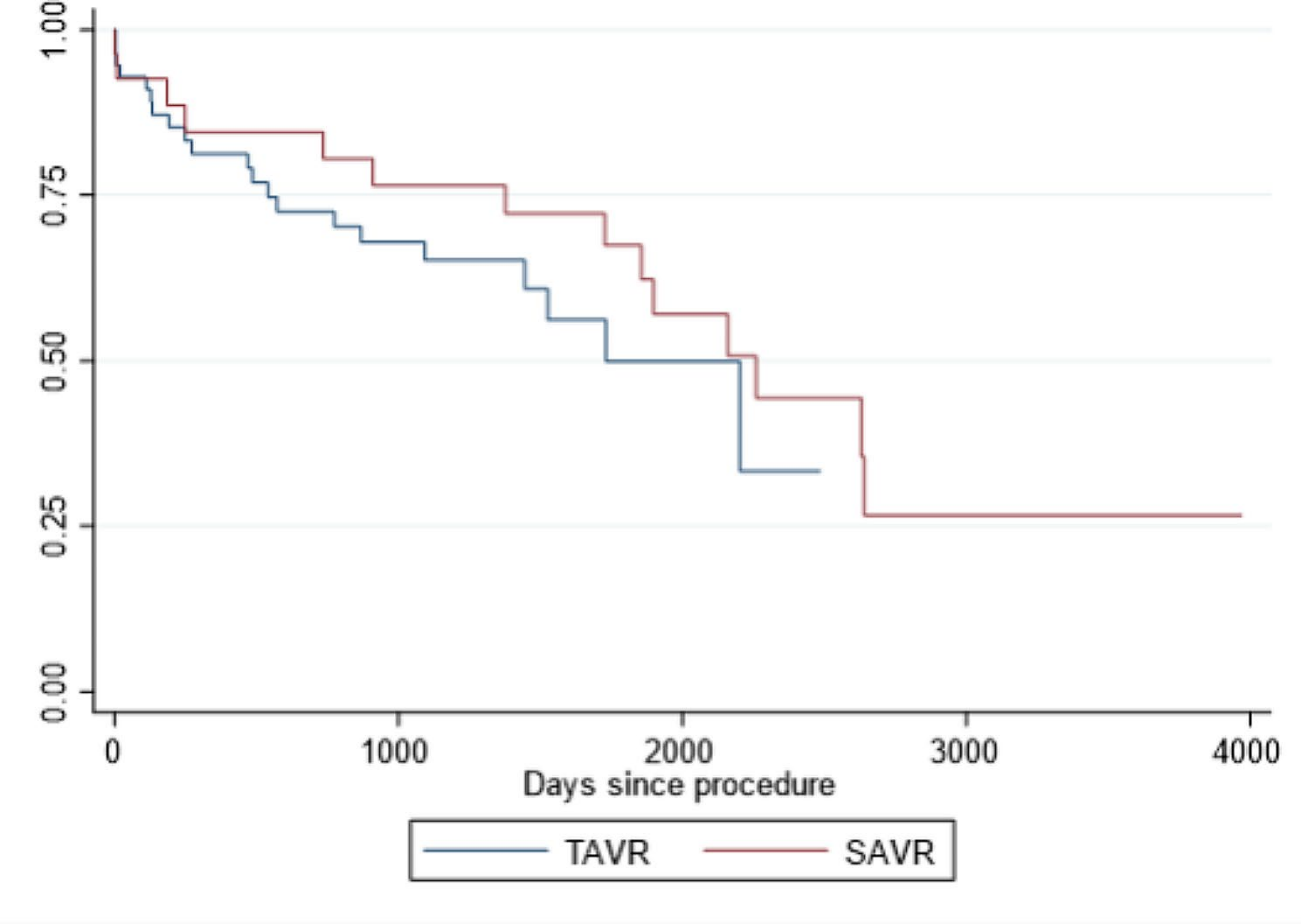
Kaplan Meier curve of overall survival following TAVR vs. SAVR, *p* = 0.3626

Six (7.2%) individuals died during the first 30 days post procedure, 4 (7.4%) TAVR and 2 (7.1%) SAVR, *p* = 0.91. A total of 35 individuals (36%) died at time of last known follow-up; 21(60%) TAVR and 14(40%) SAVR. Kaplan Meier survival curve was created and there was no significant mortality difference between TAVR and SAVR (*p* = 0.36) (Fig. 2).

When assessing predictors of 30-day mortality, one-week post procedure LVEF trended toward, but did not reach significance (OR 1.07 [95% CI, 0.99–1.16]; *p* = 0.083).

Preprocedural septal thickness and posterior wall thickness were associated with 30-day mortality (*p* = 0.006 and *p* = 0.029), however when adjusting for TAVR vs. SAVR these did not reach statistical significance (Odds ratio of 19.3 [(95% CI, 0.71-526.25]; *p* = 0.079). No other preprocedural echocardiogram measures were associated with 30-day mortality.

## Discussion

The primary results of this study are as follows: (1) There was a significant and rapid improvement in LVEF following AVR. (2) LVEF continued to increase up to two years post-procedure. (3) There was no significant difference in mean EF recovery between TAVR and SAVR. (4) There was no significant difference in mortality between TAVR and SAVR patients.

Multiple RCTs have looked at 1 year mortality in patients with EF > 20% and demonstrated the noninferiority of TAVR to SAVR regardless of surgical risk [4–7]. Severely reduced EF is a known risk factor for surgical intervention, however, there is conflicting evidence about the effect of reduced LVEF on TAVR mortality because the majority of RCT excluded LVEF < 20% [4, 8, 15, 16]. Those trials that have included severely reduced EF had average LVEF over 55% [6, 7]. No prior studies have addressed mortality and EF recovery in patients with severely reduced LVEF (< 25%).

The decision of AVR method is currently based on presumed perioperative risk and possible long-term complications extrapolated from studies with higher baseline EF. Our study confirms previously seen relationships between mortality and AVR method in patients with severely reduced EF. In addition, it adds newly discovered findings of progressive LVEF recovery greater than two years post-procedure.

Data from PARTNER 1 trial [16] and Baron et al. [14] found no association between LVEF and TAVR mortality [11]. A later analysis of PARTNER 2 trial [8] found LVEF < 50% to be an independent risk factor for mortality and concordant findings were seen in several meta-analyses [17, 18]. While reduced EF was associated with increased mortality, this relationship did not persist when separating EF < 40% versus < 50% [8, 18]. Schaefer et al. [19] looked at patients with severely reduced EF and found a two-fold increase in mortality in patients with LVEF < 30% as compared to LVEF > 30% following TAVR.

Our study demonstrated no significant difference in short term (30-day) or long-term mortality rates between the TAVR and SAVR, however, was limited due to small sample size.

Both groups had a significant and rapid improvement in LVEF following AVR. EF increased on average by 60% at one week post procedure. Subgroup analysis by Dauerman and colleagues [20] found a similar rapid increase in LVEF (> 10%) within 48 h after TAVR in patients with EF > 20%. In our study, the EF increased in both groups until final follow-up (> 24 months post procedure). At final follow-up the average LVEF was double (41.26) the pre-procedural LVEF (20.48). Baron et al. [21] suggested patients with reduced EF may have a more significant rise in LVEF following AVR. However, no other study has shown such a dramatic increase in EF that continued until greater than two years post procedure [4, 6, 19, 21]. This suggests a more robust response in patients with severely reduced EF.

The rate of myocardial infarctions was higher in the TAVR group (37.5%) compared to the SAVR group (11.1%) but did not reach statistical significance. Myocardial infarction is known to decrease myocardial viability, and this variable could diminish expected EF recovery post-AVR.

One week post procedure LVEF trended toward but did not reach significance as a predictor of 30-day mortality. Dauerman et al. [20] showed similar findings with a trend of increased mortality in patients that were slow to recover EF that was not statistical significance. Subgroup analysis of the PARTNER trials by Kolte et al. [22] found that patients with reduced LVEF (< 50%) and rapid increase in LVEF by 30-days post procedure had improved one year mortality. This suggests post procedure LVEF may be useful as a prognostic indicator for mortality.

## Limitations

The retrospective and single institution nature of this study is associated with inherent bias, which limits its generalizability. The study is significantly limited by the small sample size. Many patients did not have echocardiogram at predetermined time intervals and some echocardiogram parameters were not reported at each assessed time interval. The TAVR subgroup at baseline had more comorbidities and prior interventions deeming them a higher risk group. Subgroup analysis was performed to minimize these effects. The time frame for this study ranged from 2012 to 2020 during which techniques and medical devices for TAVRs advanced rapidly and could have effected outcomes. Lastly, this study did not review stress echocardiograms to determine contractile reserve and LV diastolic function.

## Conclusion

Among patients with severe aortic stenosis and markedly reduced LVEF (< 25%) there was no significant difference in 30-day mortality following TAVR versus SAVR. Both groups had a similar rapid increase in postprocedural LVEF and average LVEF continued to increase at subsequent follow-ups. At final follow-up (> 24 months) LVEF had doubled. Further evaluation with large multicentered investigation is recommended.

## Data Availability

The datasets used/or analyzed during the current study are available from the corresponding author on reasonable request.

## Abbreviations

AS: Aortic stenosis
AVR: Aortic valve replacement
SAVR: Surgical aortic valve replacement
TAVR: Transcatheter aortic valve replacement
LV: Left ventricular
LVEF: left ventricular ejection fraction
CABG: Coronary artery bypass graft
ICD: Implantable cardiac device
TEE: Transesophageal echocardiogram
TTE: Transthoracic echocardiogram
